# Health Tract, No. 135

**Published:** 1865

**Authors:** 


### HEALTH TRACT, No. 135.

### CURES.

Instead of all the fools being dead, we verily believe they are on the increase, in spite of our ten years' labor in the endeavor to wedge a little mite of common-sense into the craniums of Tom, Dick, and Harry. When in England some years ago, we thought patent medicines and secret remedies had quite as great a run as in America, although England had had nearly two thousand years' longer schooling than we. This would seem to prove that the more intelligent a community becomes, the more gullible it grows. In looking over our exchanges, religious and otherwise, it is perfectly clear, according to the affidavits and testimonials of clergymen, divinity doctors and doctors of law, of men and women, old grannies and maids, that every thing can be cured, from a finger-scratch to amaurosis, malignant tubercle and death-rattles, in little or no time; and that if any body dies, it is their own fault entirely. Recently, a sub-editor went to an eye-doctor.

"What's the matter with my eye?"

"Amaurosis."

"Can you cure it?"

"Oh! yes."

"How long?"

"Two weeks."

"How much?"

"You can pay five hundred dollars now, on account, and further, according to circumstances."

The quill man declined; went to Chicago, took a few warm baths, and after paying some attention to the general health, returned to New-York, apparently well of —"amaurosis!" one of the most certainly fatal of all diseases.

While all this is going on in New-York, "in the way of trade," the unprofessional "put in an oar" every now and then, free gratis for nothing. The latest thing of the kind appeared in the columns of that staid and sterling paper, the New-York *Observer*. Some writer, itching to deliver himself of an idea "as clear as mud," literally, writes to say that he is a firm believer in the "mud cure" of hydrophobia, as he knew a man who was bitten by a mad dog; a lump of mud was plastered over the wound for half a day, and at the end of thirty years, the man was living in good health. The utter folly of putting forth such miserable stuff as this, in reference to so serious, so terrible a thing as hydrophobia, may be seen at once, in the fact that John Hunter, than whom there has never yet lived a greater surgeon, says he knew twenty-one persons who had been bitten by mad dogs, and but one of the whole number became hydrophobic. Each of the twenty might have claimed that his was a "cure." It is the fashion now to call every sore throat a child has, "diphtheria," and every child that gets well was cured by the thing which was done for it; but the next person who tries it, loses his child, which might have been saved by promptly calling in medical advice. No doubt the virtues of the "mad stone" have grown out of the fact, that now and then persons who have been bitten by mad dogs, or supposed to be rabid, have remained unharmed after the application of the stone; not because of any virtue it possessed of antagonizing the poison, but simply because the system of the bitten individual was not at the time susceptible to the influences of the virus. A child *said* to have diphtheria gets well after smoking tar, poured on a live coal in the bowl of a common pipe, or by stretching a bag of ashes and salt, or mush and molasses, from ear to ear under the jaw; but to say that these are cures of the terrible complaint, is the lamest of all conclusions. No business man would risk five dollars on that kind of reasoning. And yet it is upon such grounds that the papers are filled with "cures," *certain*, *infallible*, of every malady under the sun. By all that is sacred in a holy human life, we urge the reader, when he or any of his are ailing in any way whatever, to do one of two things; either do nothing, and let nature take care of herself, or consult your family physician, who, if educated to his profession, will take an interest in you beyond any stranger; or, if he sees the case is beyond his skill, will frankly acknowledge it, and will take pains to turn you over to some man of eminence and acknowledged ability

